# Preparation, Hydration Characteristics, and Carbon Footprint Assessment of Sulfoaluminate Cement Prepared by Co-Utilization of Industrial Solid Wastes and Bauxite

**DOI:** 10.3390/ma19102122

**Published:** 2026-05-18

**Authors:** Yanzhou Peng, Xiaohang Miao, Dejun Gao, Chunhu Fan

**Affiliations:** 1 Hubei Provincial Key Laboratory of Disaster Prevention and Mitigation, China Three Gorges University, Yichang 443002, China; 2 College of Civil Engineering and Architecture, China Three Gorges University, Yichang 443002, China; 202308140021028@ctgu.edu.cn (X.M.); 202108590021141@ctgu.edu.cn (C.F.)

**Keywords:** sulfoaluminate cement, phosphogypsum, calcium carbide residue, calcination regime, strength, hydration, carbon footprint

## Abstract

This study evaluates the technical feasibility, environmental sustainability, and economic viability of producing sulfoaluminate cement (SW-SAC) by co-utilizing bauxite and industrial solid wastes—phosphogypsum, calcium carbide residue (CCR), and red mud—with the solid wastes accounting for approximately 75% of the raw meal. CCR replaces limestone as the primary CaO source, releasing H_2_O instead of CO_2_, while phosphogypsum supplies SO_3_; the raw meal is directly calcined in a single step at 1300–1350 °C, 100–150 °C below that of ordinary Portland cement (OPC). Calcination temperature and holding time were optimized through phase analysis, microstructural observation, free lime (f-CaO) determination, and strength testing. SW-SAC meeting the 42.5 strength class was then prepared using phosphogypsum as a setting regulator and phosphorus slag or limestone powder as Supplementary materials. X-ray diffraction (XRD), thermogravimetry (TG), and scanning electron microscopy (SEM) were used to examine hydration products and microstructural evolution. The optimized clinker was dominated by ye’elimite ($C_{4}A_{3}\bar{S}$) and belite (C_2_S). Phosphorus slag favored the formation of gel-like products at later ages, whereas limestone powder promoted ettringite (AFt) stabilization and monocarboaluminate (Mc) formation. SW-SAC exhibited a lower carbon footprint than both Type P·I Portland cement and conventional SAC, and a lower production cost than conventional SAC. These results demonstrate a promising low-carbon route for high-value utilization of industrial solid wastes.

## 1. Introduction

Portland cement is the most widely used construction material worldwide; yet its manufacture is increasingly scrutinized because it is both energy- and carbon-intensive. The cement industry is estimated to account for approximately 7–8% of global anthropogenic CO_2_ emissions [1]. As the world’s largest cement producer, China reported a cement output of 1.825 billion tons in 2024, associated with CO_2_ emissions of 1.095 billion tons [2]. Against the backdrop of China’s “dual-carbon” targets (carbon peaking and carbon neutrality), reducing energy consumption and CO_2_ emissions in the cement sector has become a pressing requirement for green transformation and long-term sustainability.

Sulfoaluminate cement (SAC) has attracted sustained interest due to its rapid hardening, high early strength, relatively low shrinkage, and strong resistance to sulfate attack [3,4,5,6,7]. Moreover, the clinker calcination temperature of SAC (1250–1350 °C) [8,9,10] is significantly lower than that of ordinary Portland cement (OPC) clinker (≈1450 °C) [11] which effectively reduces energy consumption and CO_2_ emissions during firing, positioning SAC as a promising low-carbon binder for the cement industry [12,13]. Nevertheless, conventional SAC production relies heavily on high-grade bauxite [14,15] and limestone as principal raw materials. The limited reserves and supply constraints of these natural resources constitute a practical barrier to the large-scale deployment of SAC, underscoring the need for alternative raw-material systems that lessen dependence on virgin mineral resources.

Meanwhile, accelerating industrialization has led to the continuous accumulation of industrial by-products, many of which remain underutilized, resulting in resource losses and mounting environmental burdens. In China, annual phosphogypsum generation is approximately 75 million tons, whereas the comprehensive utilization rate is only about 40% [16]. Calcium carbide residue exceeds 28 million tons per year, with a utilization rate below 30% [17]. Red mud production approaches 100 million tons annually, yet its effective utilization remains below 5% [18]. Long-term stockpiling of these by-products occupies substantial land resources and prevents the recovery of valuable mineral constituents, thereby posing potential risks to the ecological environment [19].

Importantly, these industrial by-products contain the key oxides required for SAC clinker formation, including CaO, Al_2_O_3_, SiO_2_, Fe_2_O_3_, and SO_3_, suggesting their theoretical suitability as cement raw materials. Accordingly, both domestic and international studies have explored the synthesis of SAC clinker via the synergistic utilization of multiple solid wastes. Gao et al. [20] prepared high-belite sulfoaluminate cement by direct calcination at 1300 °C for 60 min using red mud, blast furnace slag, steel slag, desulfurization gypsum, and calcium carbide residue. Rungchet et al. [21] synthesized belite sulfoaluminate cement through a hydrothermal–calcination route based on fly ash, aluminum-rich sludge, and desulfurization gypsum, achieving a 1-day compressive strength of 16 MPa. Bai et al. [22] produced belite–calcium sulfoaluminate–ternesite (BYT) clinker from coal gangue, calcium carbide residue, and desulfurization gypsum. Collectively, these studies indicate that SAC clinker can be synthesized in the 1250–1350 °C range while meeting relevant strength requirements, thereby offering an energy-saving advantage relative to OPC.

Phosphogypsum has received particular attention as a sulfate-bearing component for SAC systems. During clinker firing, soluble fluorine in phosphogypsum can react to form CaF_2_, which acts as a mineralizer by lowering the liquid-phase formation temperature and promoting the formation of key clinker phases [23]. In this context, various routes have been proposed to substitute natural gypsum with phosphogypsum. Pre-decomposition treatment has been reported to improve cement performance, although it typically involves a more complex process and higher energy demand [24,25,26]. By contrast, direct incorporation of phosphogypsum into the raw meal has been explored as a more streamlined and cost-effective route [27,28,29]. Shen et al. [30] directly replaced natural gypsum with phosphogypsum in the raw mix and produced SAC clinker by calcining at 1250–1300 °C for 30 min, reporting a phosphogypsum decomposition ratio of up to 36%. Kramar et al. [31] calcined a blend containing 70 wt% marl, 10 wt% bauxite, and 20 wt% phosphogypsum at 1320 °C to obtain belite–sulfoaluminate cement with a compressive strength of 45.9 MPa, further supporting the feasibility of phosphogypsum utilization in SAC clinker production.

Calcium carbide residue is another attractive CaO-rich calcareous source. Its main component, Ca(OH)_2_, dehydrates at temperatures much lower than the decarbonation temperature of limestone, enabling dehydration at relatively low temperatures and potentially reducing calcination energy demand. Moreover, the absence of carbonates avoids CO_2_ release from decarbonation, which can further lower process emissions. Previous studies have consistently shown that calcium carbide residue can fully replace limestone in clinker production, delivering both industrial waste valorization and combined energy- and carbon-saving benefits [8,22,32,33]. However, some phosphogypsum-based SAC routes still use phosphogypsum as a major CaO-bearing raw material or combine phosphogypsum with limestone in the raw meal [34,35], while others require prior thermal treatment of phosphogypsum before clinker production [36]. These approaches leave room for further simplifying the raw material design and calcination process while reducing CO_2_ emissions from calcareous components.

In light of the above, this study systematically investigates the feasibility of producing solid-waste-derived sulfoaluminate cement (SW-SAC) using phosphogypsum, calcium carbide residue, red mud, and bauxite, with industrial solid wastes accounting for approximately 75% of the raw meal. In this work, calcium carbide residue was used as the main CaO source instead of limestone, while phosphogypsum was mainly used to supply SO_3_. This raw-material combination enabled direct single-step calcination at 1300–1350 °C, which is 100–150 °C lower than the typical clinkerization temperature of OPC. On the basis of the theoretical design of the target clinker mineralogy and the raw-meal calculations, the effects of calcination temperature and holding time on clinker phase assemblage and performance were examined to identify an optimal calcination regime. Subsequently, phosphogypsum was used as a setting retarder, and either phosphorus slag or limestone powder was incorporated as a blended component to produce SW-SAC meeting the 42.5 strength class requirement. The hydration and hardening behavior was then elucidated using X-ray diffraction (XRD), thermogravimetry–derivative thermogravimetry (TG–DTG), and scanning electron microscopy (SEM), and a life cycle assessment (LCA) [37] was conducted to quantify the carbon footprint of SW-SAC production, together with a production cost analysis, in comparison with ordinary Portland cement (OPC; denoted as P·I in the Chinese standard) and conventional SAC, thereby evaluating its environmental and economic performance as a potential low-carbon binder [38].

## 2. Materials and Experimental Methods

### 2.1. Raw Materials

**Figure 1:**
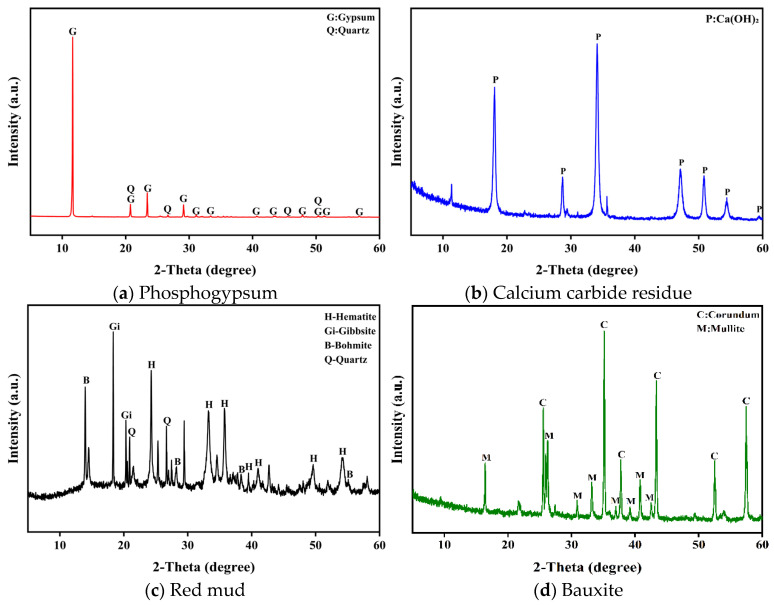
XRD patterns of phosphogypsum, calcium carbide residue, red mud, and bauxite used in this study.

Phosphogypsum (PG) and calcium carbide residue (CCR) were supplied by Hubei Yihua Group Co., Ltd. (Yichang, China). Red mud (RM) was obtained from Hebei Daye Mineral Products Processing Plant (Shijiazhuang, China), and bauxite (BX) was provided by Hebei Yu’er Environmental Protection Technology Co., Ltd. (Shijiazhuang, China). Granulated electric-furnace phosphorus slag (PS) and limestone powder (LP) were sourced from Hubei Yatai Chemical Co., Ltd. (Yichang, China). The major oxide compositions of the raw materials were determined by X-ray fluorescence (XRF), and the results are summarized in Table 1. The X-ray diffraction (XRD) patterns of the raw materials are presented in Figure 1.

### 2.2. Design of SW-SAC Clinker Mineral Composition and Raw Meal Proportioning

As summarized in Table 2, the target phase assemblage of the SW-SAC clinker was specified according to the in-tended mineralogical composition. The corresponding theoretical oxide composition was derived from the phase targets using Equations (1)–(5), and the alkalinity coefficient (*C_m_*), alumina-to-sulfur ratio (*P*), and alumina-to-silicon ratio (N) were calculated using Equations (6)–(8). These indices were subsequently used as constraints in the mass-balance calculations to determine the raw meal proportions. The resulting values of the indices, together with the designed raw meal proportions, are listed in Table 2 [10,28,37]. Cm quantifies the extent to which the CaO supplied by the raw meal meets the CaO demand required for forming the target clinker phases; therefore, Cm was maintained below 1.0 to avoid excessive free lime. The alumina-to-sulfur ratio P assesses whether the available Al_2_O_3_—after accounting for that consumed in ferrite formation—is sufficient to react with CaSO_4_ to form $C_{4}A_{3}\bar{S}$; accordingly, P was limited to below 3.82. In contrast, N mainly describes the phase proportion between $C_{4}A_{3}\bar{S}$ and C_2_S in the clinker. Because tuning this ratio was not an objective of the present study, *N* is reported for completeness but was not controlled.


(1) $$\omega \left(\mathrm{CaO}\right)=0.6512\omega \left(C_{2}S\right)+0.3672\omega \left(C_{4}A_{3}\bar{S}\right)+0.4616\omega \left(C_{4}\mathrm{AF}\right)+0.4119\omega \left({\mathrm{CaSO}}_{4}\right)$$

(2) $$\omega \left({\mathrm{SiO}}_{2}\right)=0.3488\omega \left(C_{2}S\right)$$

(3) $$\omega \left({\mathrm{Al}}_{2}O_{3}\right)=0.5016\omega \left(C_{4}A_{3}\bar{S}\right)+0.2098\omega \left(C_{4}\mathrm{AF}\right)$$

(4) $$\omega \left({\mathrm{Fe}}_{2}O_{3}\right)=0.3286\omega \left(C_{4}\mathrm{AF}\right)$$

(5) $$\omega \left({\mathrm{SO}}_{3}\right)=0.1312\omega \left(C_{4}A_{3}\bar{S}\right)+0.5881\omega \left({\mathrm{CaSO}}_{4}\right)$$

(6) $$C_{m}=\frac{\omega \left(\mathrm{CaO}\right)-0.7\omega \left({\mathrm{TiO}}_{2}\right)}{0.73\left[\omega \left({\mathrm{Al}}_{2}O_{3}\right)-0.64\omega \left({\mathrm{Fe}}_{2}O_{3}\right)\right]+1.4\omega \left({\mathrm{Fe}}_{2}O_{3}\right)+1.87\omega \left({\mathrm{SiO}}_{2}\right)}$$

(7) $$P=\frac{\omega \left({\mathrm{Al}}_{2}O_{3}\right)-0.64\omega \left({\mathrm{Fe}}_{2}O_{3}\right)}{\omega \left({\mathrm{SO}}_{3}\right)}$$

(8) $$N=\frac{\omega \left({\mathrm{Al}}_{2}O_{3}\right)-0.64\omega \left({\mathrm{Fe}}_{2}O_{3}\right)}{\omega \left({\mathrm{SiO}}_{2}\right)}$$

### 2.3. Clinker Calcination Process and Cement Preparation

**Figure 2:**
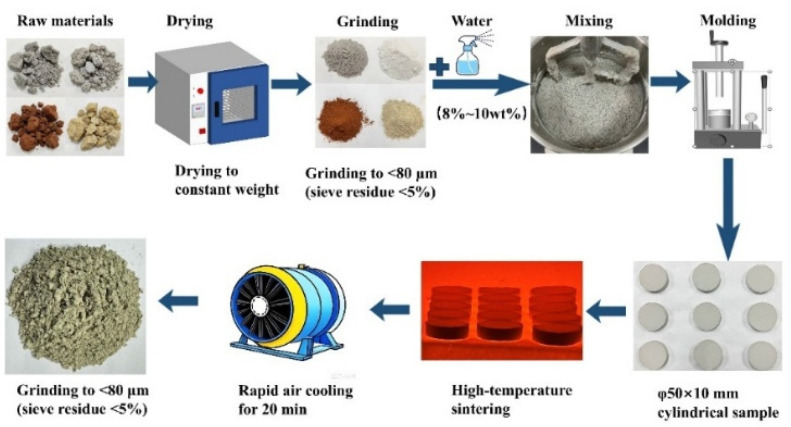
Schematic illustration of the clinker calcination procedure.

After drying, the raw materials were ball-milled to pass an 80 μm sieve and dry-blended according to the proportions listed in Table 2. The dry blend was spray-wetted to facilitate pelletization and pressed into pellets (Ø50 mm × 10 mm) with a moisture content of 8–10%. The pellets were calcined in a high-temperature furnace (Shanghai Jujing Precision Instrument Manufacturing Co., Ltd., Shanghai, China). For temperature screening, the furnace was heated at 5 °C/min to 1300, 1325, 1350, 1375, or 1400 °C and held for 30 min. The holding time was subsequently varied from 10 to 50 min (10, 20, 30, 40, or 50 min) at the selected temperature. After calcination, the pellets were rapidly fan-cooled to room temperature, mechanically crushed, and milled. The resulting clinker was sieved to <80 μm, with a residue below 5%, yielding the SW-SAC clinker. A schematic of the preparation procedure is shown in Figure 2.

A two-stage batching optimization was subsequently performed. In the first stage, phosphogypsum was added at different dosages based on the gypsum coefficient M, calculated using Equation (9), which governs the type of hydration products associated with $C_{4}A_{3}\bar{S}$ and the AFm/AFt assemblage [37,39]. Specifically, AFm is expected to dominate when M ≤ 1; AFm and AFt typically coexist for 1 < M < 2; and AFt becomes predominant when M ≥ 2. Accordingly, phosphogypsum dosages corresponding to M = 0, 0.7, 1.1, 1.5, and 1.9 were selected and tested as setting regulators (Table 3). After the optimal phosphogypsum dosage was identified, the second stage used the F13 formulation as the baseline, with phosphorus slag or limestone powder proportionally replacing the clinker and phosphogypsum at 0, 5, 10, 15, and 20 wt% to prepare blended SW-SAC. The optimal replacement level of the supplementary material was determined experimentally (Table 4).

(9) $$M=\frac{C_{G}\times \bar{S}}{0.13\times A_{c}}$$

where *C_G_* is the mass ratio of phosphogypsum to clinker, *A_C_* is the mass fraction of ye’elimite ($C_{4}A_{3}\bar{S}$) in the clinker, and S^−^ is the mass fraction of SO_3_ in phosphogypsum. The constant 0.13 is derived from the stoichiometric relationship of ye’elimite hydration.

### 2.4. Experimental Methods

#### 2.4.1. Physicomechanical Properties of SW-SAC Clinker and Cement

Standard consistency water demand, setting time, and soundness were determined in accordance with GB/T 1346—2024 [40]. Given the rapid hydration of SAC, the paste mixing procedure followed JC/T 2282—2014 [41], using high-speed mixing for 40 s. The f-CaO content of the clinker was determined by the ethylene glycol method in accordance with GB/T 176-2017 [42]. The compressive strength of clinker paste was measured using 20 mm × 20 mm × 20 mm specimens at a water-to-binder ratio (w/b) of 0.30; specimens were demolded after 6 h and cured at (20 ± 2) °C with a relative humidity of at least 95%. Cement mortar strength was tested following GB/T 17671-2021 [43] with a w/b of 0.44; specimens were demolded after 24 h and then cured in water at (20 ± 2) °C. All specimens were tested at 1, 3, and 28 days.

#### 2.4.2. Mineralogical Analysis of SW-SAC Clinker

Rietveld quantitative phase analysis (RQPA) was performed on the XRD data of the clinker using X’Pert HighScore Plus software (version 5.2, Malvern Panalytical, Almelo, The Netherlands) to obtain the mass fractions of the crystalline phases [44,45]. The crystallographic information used for RQPA is provided in Table 5.

#### 2.4.3. Characterization of Clinker and Hydration Products

The chemical composition of the SAC clinker was determined by X-ray fluorescence (XRF) using a Shimadzu EDX-7000 spectrometer (Shimadzu Corporation, Kyoto, Japan). The samples were prepared as pressed pellets (40 mm in diameter and 5 mm in thickness) and measured with an Rh target and a Be window (0.75 μm) at a tube voltage of 40 kV and a tube current of 80 mA. The mineralogical phases of the clinker and hydrated samples were qualitatively identified by X-ray diffraction (XRD) using a Rigaku SmartLab (9 kW) diffractometer (Rigaku Corporation, Tokyo, Japan). Measurements were performed with Cu Kα radiation (λ = 1.5406 Å) at 40 kV and 30 mA over a 2θ range of 3–70° with a step size of 0.02°. Thermogravimetric analysis (TGA) of the hydrated samples was conducted using a NETZSCH STA 449 F5 simultaneous thermal analyzer (NETZSCH-Gerätebau GmbH, Selb, Germany). The measurements were carried out under flowing nitrogen (100 mL/min), heating from 30 °C to 1050 °C at 10 °C/min. The microstructures of the clinker and hydrated samples were characterized by scanning electron microscopy (SEM) using a JSM-IT800 scanning electron microscope (JEOL Ltd., Tokyo, Japan).

## 3. Results and Discussion

### 3.1. Effects of Calcination Temperature and Holding Time on SW-SAC Clinker Performance

#### 3.1.1. Effect of Calcination Temperature

**Figure 3:**
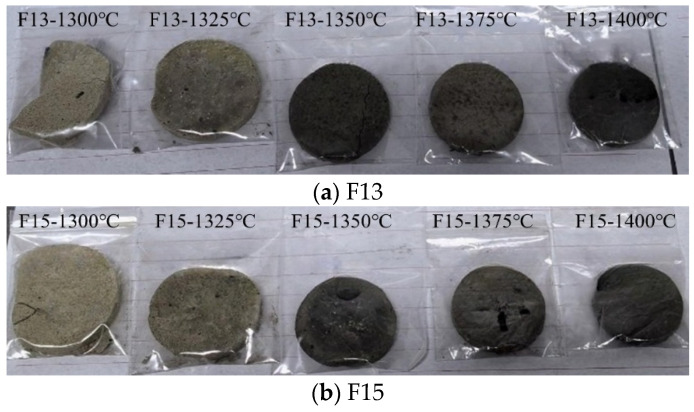
Appearance of clinkers prepared at different calcination temperatures.

The raw meals corresponding to the F13 and F15 formulations (Table 2) were calcined at 1300, 1325, 1350, 1375, and 1400 °C, held for 30 min, and then rapidly fan-cooled. The resulting clinkers are shown in Figure 3. At 1300–1325 °C, the clinker appeared light gray and showed slight disintegration. Increasing the calcination temperature led to a progressive darkening of color—from dark gray (1325 °C) to brown (1350 °C) and deep black (1375 °C)—accompanied by a modest shrinkage in volume and a visibly denser structure. Although minor surface cracking was observed, powdering was no longer evident, indicating normal sintering behavior. These macroscopic changes suggest that higher temperatures promote more complete solid-state reactions and progressively improve sintering during clinker formation.

**Figure 4:**
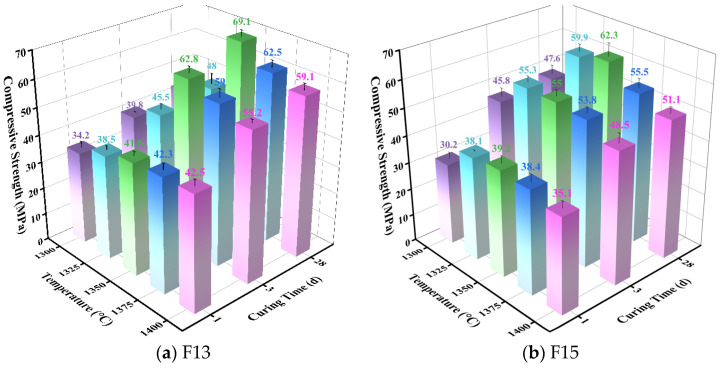
Compressive strength of clinkers produced from the F13 and F15 raw meals calcined at different temperatures.

**Figure 5:**
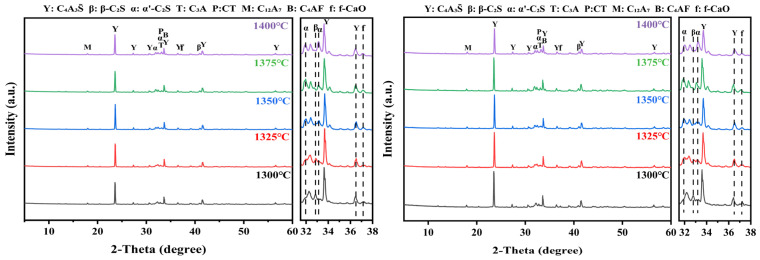
XRD patterns of F13 and F15 clinkers calcined at different temperatures.

Figure 4 presents the compressive strength results of the clinkers produced from the F13 and F15 raw meals calcined at different temperatures. For both formulations, compressive strength increased with temperature up to an optimum and then declined at higher temperatures. For F13, the 1 d compressive strength of the clinker calcined at 1350 °C was 41.6 MPa, marginally lower than those obtained at 1375 °C (42.3 MPa) and 1400 °C (42.5 MPa). This small early-age difference did not persist: the 3 d and 28 d strengths at 1350 °C were clearly higher than the corresponding values at the other temperatures, with the 28 d strength reaching 69.1 MPa. For F15, early-age strength (1 and 3 d) at 1350 °C differed only slightly from that at the other temperatures; however, the 28 d strength increased to 62.3 MPa, outperforming all other calcination conditions. The corresponding XRD patterns are provided in Figure 5 and discussed below.

Figure 5 shows the XRD patterns of clinkers produced from the F13 and F15 raw meals calcined at different temperatures. Regardless of calcination temperature, the clinkers developed a mineral assemblage dominated by ye’elimite ($C_{4}A_{3}\bar{S}$) and belite (C_2_S), with minor amounts of C_4_AF, C_12_A_7_, and C_3_A also detected. Based on the XRD data, Rietveld quantitative phase analysis (RQPA) was performed, and the phase contents of the F13 and F15 clinkers are listed in Table 6 and Table 7.

Table 6 and Table 7 show that the prepared clinkers contained a higher fraction of ye’elimite ($C_{4}A_{3}\bar{S}$) than the theoretical design, while the C_4_AF content was lower than the target value reported in Table 2. Ye’elimite occurred as both orthorhombic and cubic polymorphs, with the orthorhombic form predominating. This can be rationalized by Fe^3+^ introduced via red mud, which partially substitutes for Al^3+^ in the sulfoaluminate lattice and thereby favors the formation of a cubic anhydrous calcium sulfoaluminate phase, Ca_4_(A,F)_3_S [46]. As calcination temperature increased, the ye’elimite content in both F13 and F15 first rose and then declined, reaching maxima at 1350 °C (64.06% and 59.96%, respectively), whereas the amounts of C_3_A and C_12_A_7_ generally increased with temperature. Such evolution suggests that, above 1350 °C, ye’elimite partially decomposes (Equation (10)) to yield CA and CaO [47], which can subsequently react to form C_12_A_7_ according to Equation (11). In the presence of free lime (f-CaO), part of the C_12_A_7_ may further react with CaO to generate C_3_A (Equation (12)) [43,44,48]. Taken together, the reduction in ye’elimite at elevated temperature is consistent with its thermal decomposition, while the accompanying increase in fast-hydrating calcium aluminate phases (CA, C_3_A, and C_12_A_7_) may impede early densification of the hardening clinker paste, offering a plausible explanation for the strength loss observed when the calcination temperature exceeds 1350 °C (Figure 4).

(10) $$2(3\mathrm{CaO}\cdot 3{\mathrm{Al}}_{2}O_{3}\cdot {\mathrm{CaSO}}_{4})\to 6(\mathrm{CaO}\cdot {\mathrm{Al}}_{2}O_{3})+2\mathrm{CaO}+2{\mathrm{SO}}_{2}+O_{2}$$

(11) $$7(\mathrm{CaO}\cdot {\mathrm{Al}}_{2}O_{3})+5\mathrm{CaO}\to 12\mathrm{CaO}\cdot 7{\mathrm{Al}}_{2}O_{3}$$

(12) $$\left(12\mathrm{CaO}\cdot 7{\mathrm{Al}}_{2}O_{3})+9\mathrm{CaO}\to 7(3\mathrm{CaO}\cdot {\mathrm{Al}}_{2}O_{3}\right)$$

**Figure 6:**
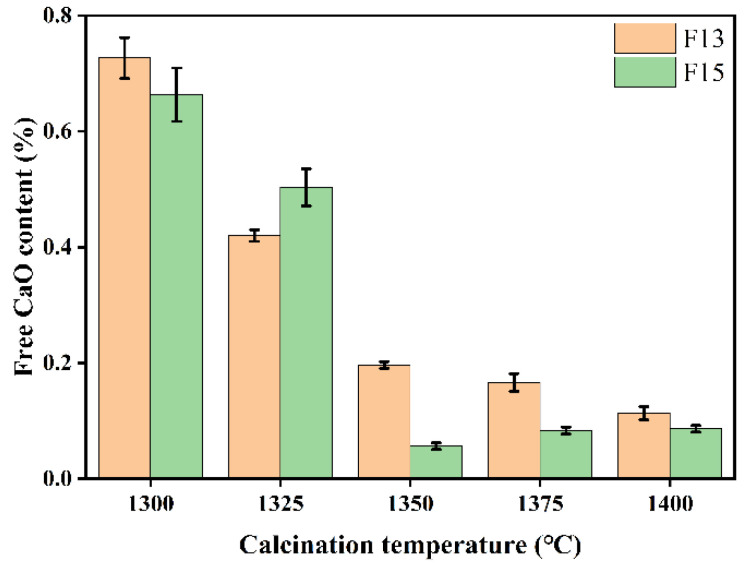
f-CaO content of F13 and F15 clinkers calcined at different temperatures.

Figure 6 presents the measured free lime (f-CaO) contents in the F13 and F15 clinkers calcined at different temperatures. In both series, f-CaO decreased progressively as the calcination temperature increased. At 1300 °C, the f-CaO contents were 0.73% (F13) and 0.66% (F15). Raising the temperature to 1350 °C reduced these values to 0.20% and 0.06%, respectively, whereas further increases to 1375–1400 °C caused little additional change and kept f-CaO below 0.20% in both clinkers. This trend reflects incomplete reactions at 1300 °C, where part of the CaO had not yet been incorporated into clinker phases and therefore persisted as free lime. Once the temperature reached 1350 °C, solid-state reactions accelerated, consuming f-CaO and promoting the formation of clinker minerals such as ye’elimite ($C_{4}A_{3}\bar{S}$), which accounts for the pronounced decrease [49]. Above 1350 °C, partial decomposition of $C_{4}A_{3}\bar{S}$ can generate additional CaO; however, the newly formed CaO is expected to be consumed through subsequent reactions (Equations (11) and (12)) to form calcium aluminate phases, thereby maintaining f-CaO at a low level [47].

#### 3.1.2. Effect of Holding Time

**Figure 7:**
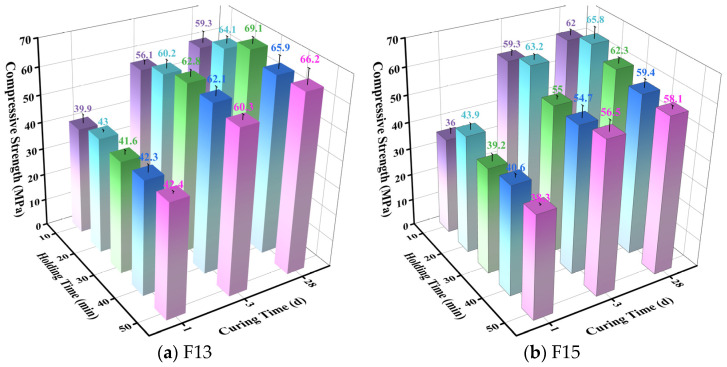
Compressive strength of F13 and F15 clinkers calcined at 1350 °C with different holding times.

**Figure 8:**
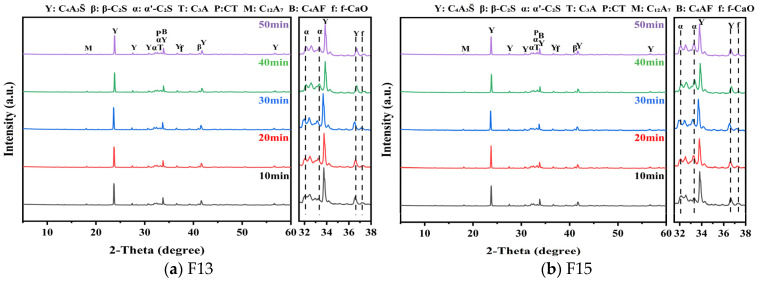
XRD patterns of F13 and F15 clinkers calcined at 1350 °C with different holding times.

The F13 and F15 raw meals were calcined at 1350 °C with holding times of 10, 20, 30, 40, and 50 min, followed by rapid air-cooling to obtain SW-SAC clinker. The influence of holding time on clinker compressive strength, phase assemblage, and free lime (f-CaO) content was then evaluated. The compressive strength results are presented in Figure 7, and the corresponding XRD patterns are provided in Figure 8. For both formulations, strength increased with holding time up to an optimum and then declined upon further extension. For F13, the best performance was achieved at 30 min, where the 3 d and 28 d strengths exceeded those of the other holding conditions and the 28 d strength peaked at 69.1 MPa. In contrast, F15 reached its optimum at 20 min, exhibiting higher strengths at all ages than the other holding times, with a maximum 28 d strength of 65.8 MPa.

The XRD results for clinkers obtained at different holding times are shown in Figure 8. The identified phases are essentially the same as those observed under varying calcination temperatures, while a slight left shift in several characteristic reflections is likely associated with subtle changes in lattice parameters [50]. Phase contents quantified by RQPA for the F13 and F15 clinkers are listed in Table 8 and Table 9. As holding time increased, the intensity of ye’elimite ($C_{4}A_{3}\bar{S}$) reflections generally rose initially and then declined, whereas the contents of C_3_A and C_12_A_7_ increased overall. Notably, the F13 clinker held for 30 min and the F15 clinker held for 20 min exhibited relatively higher $C_{4}A_{3}\bar{S}$ contents together with lower levels of C_3_A and C_12_A_7_. This evolution suggests that extending the holding time promotes partial decomposition of $C_{4}A_{3}\bar{S}$, with the decomposition products further converting to C_12_A_7_ and, in the presence of free lime (f-CaO), continuing to transform into C_3_A. Given the rapid setting and hardening associated with C_3_A and C_12_A_7_, an increased fraction of these phases may hinder early microstructural densification of the clinker paste, offering a plausible explanation for the strength decrease observed at prolonged holding times.

**Figure 9:**
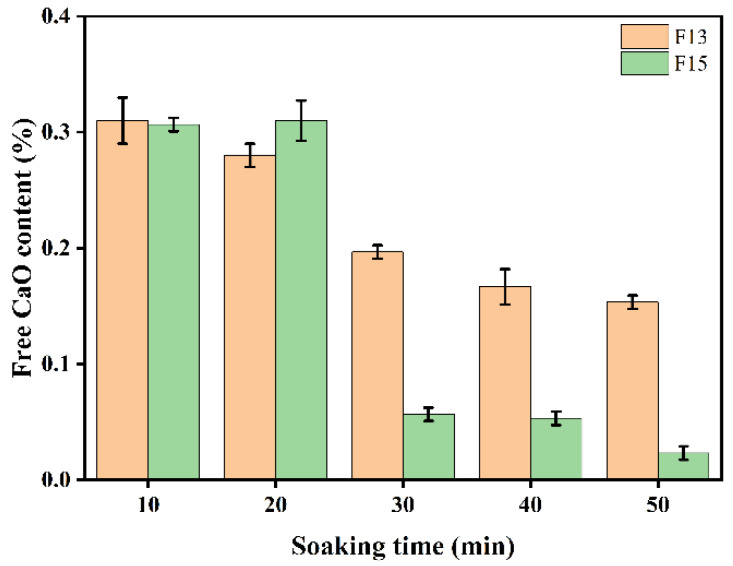
f-CaO content of F13 and F15 clinkers at different holding times.

Figure 9 shows the free lime (f-CaO) contents of clinkers with different mix designs prepared at various holding times. Extending the holding time from 10 to 50 min resulted in an overall decrease in f-CaO, with a pronounced drop at 30 min that brought the value to a relatively low level. During heating and isothermal holding, the solid-state reaction described in Equation (13) proceeds continuously, consuming f-CaO to form target phases such as ye’elimite ($C_{4}A_{3}\bar{S}$) and belite (C_2_S) [51,52]. As the system reaches 1350 °C, reaction progress increases and f-CaO decreases, remaining around 0.30% at a holding time of 10 min. At 30 min, the reactions are more complete, leading to substantial consumption of f-CaO and a marked reduction in its content; under these conditions, clinker compressive strength also attains its maximum. Prolonging the holding time to 50 min may induce partial decomposition of $C_{4}A_{3}\bar{S}$ and generate additional CaO; however, the newly formed CaO is expected to participate in subsequent reactions to form calcium aluminate phases, thereby keeping f-CaO low and relatively stable.

(13) $$3(2\mathrm{CaO}\cdot {\mathrm{Al}}_{2}O_{3}\cdot {\mathrm{SiO}}_{2})+{\mathrm{CaSO}}_{4}+3\mathrm{CaO}\;\to \;4\mathrm{CaO}\cdot 3{\mathrm{Al}}_{2}O_{3}\cdot {\mathrm{SO}}_{3}+3(2\mathrm{CaO}\cdot {\mathrm{SiO}}_{2})$$

#### 3.1.3. Mineralogical and Microstructural Analysis of SW-SAC Clinker Under Optimal Calcination Conditions

**Figure 10:**
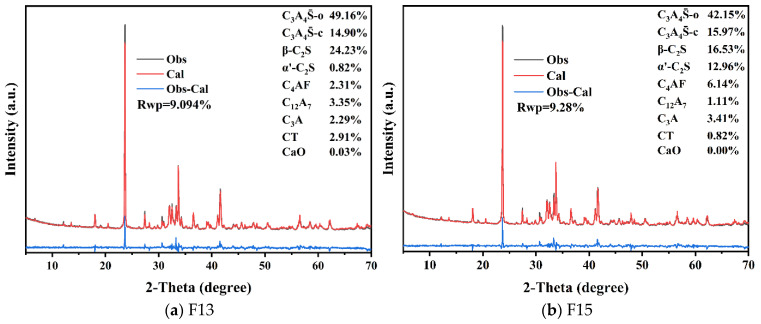
XRD–Rietveld refinement of the F13 and F15 clinkers: (**a**) F13; (**b**) F15.

Using the XRD refinement procedure described in Section 2.4.2, Rietveld quantitative analysis was performed for the F13 clinker (1350 °C, 30 min) and the F15 clinker (1350 °C, 20 min). The refinement results are shown in Figure 10.

The two clinkers exhibit broadly similar phase assemblages, dominated by ye’elimite ($C_{4}A_{3}\bar{S}$) and belite (C_2_S), with minor amounts of C_4_AF, C_12_A_7_, and C_3_A. Compared with F15, the F13 clinker contains a higher ye’elimite fraction (64.06%), whereas the C_2_S contents are comparable.

The high ye’elimite content of F13 may be attributed to two factors. First, fluorine-bearing species introduced by phosphogypsum can act as mineralizers, promoting clinker mineral formation and facilitating ye’elimite stabilization [23]. Second, Fe^3+^ originating from red mud can partially substitute for Al^3+^ in the sulfoaluminate lattice, favoring the formation of iron-bearing $C_{4}A_{3-x}F_{x}\bar{S}$, which is consistent with previous reports on Fe-bearing ye’elimite solid solutions [46].

**Figure 11:**
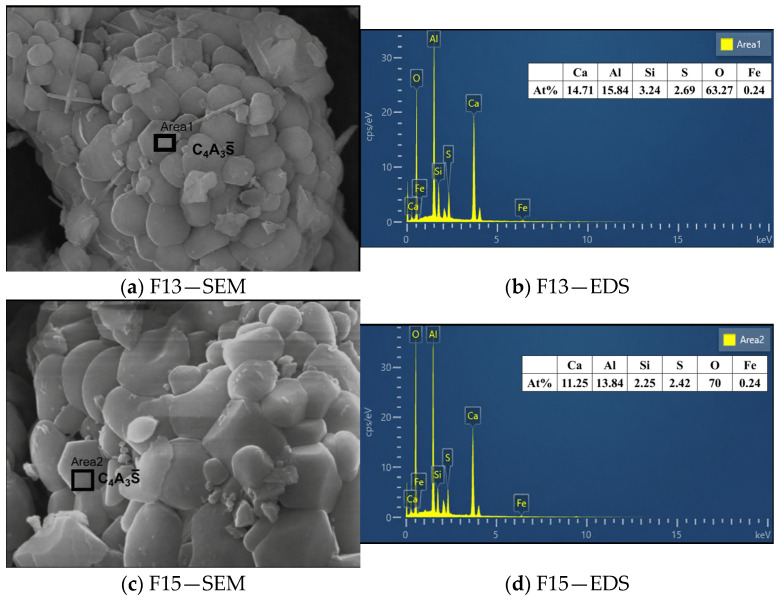
SEM micrographs and corresponding EDS spectra of F13 and F15 clinkers.

The SEM micrographs and corresponding EDS spectra of the optimized F13 and F15 clinkers are presented in Figure 11 and further confirm the identification of ye’elimite.

Figure 11 presents the SEM morphologies and EDS spectra of the F13 and F15 clinkers. The selected particles exhibit plate-like morphologies characteristic of ye’elimite. The measured Ca:Al:S atomic ratios (approximately 5.5:5.9:1 for F13 and 4.6:5.7:1 for F15) are broadly comparable to the ideal ye’elimite stoichiometry (4:6:1), which, combined with the crystal morphology, supports the identification of a ye’elimite-rich phase. In addition to the major elements, Si and Fe are detected in both spectra. The presence of Fe is attributable to the partial substitution of Al^3+^ by Fe^3+^ within the ye’elimite lattice, forming an iron-bearing solid solution $C_{4}A_{3-x}F_{x}\bar{S}$ [46,53]. Similarly, the detected Si may originate from limited Si^4+^ incorporation at the tetrahedral Al site of the sodalite-type structure, as reported for doped ye’elimite [54,55], and/or from neighboring belite (C_2_S) captured within the EDS interaction volume [56]. The incorporation of Fe into the ye’elimite structure is also consistent with the lower-than-expected C_4_AF content obtained from the Rietveld refinement of the XRD data.

### 3.2. Effects of Phosphogypsum, Phosphorus Slag, and Limestone Powder Dosages on SW-SAC Strength

#### 3.2.1. Effect of Phosphogypsum Dosage (Setting Regulator) on SW-SAC Performance

**Figure 12:**
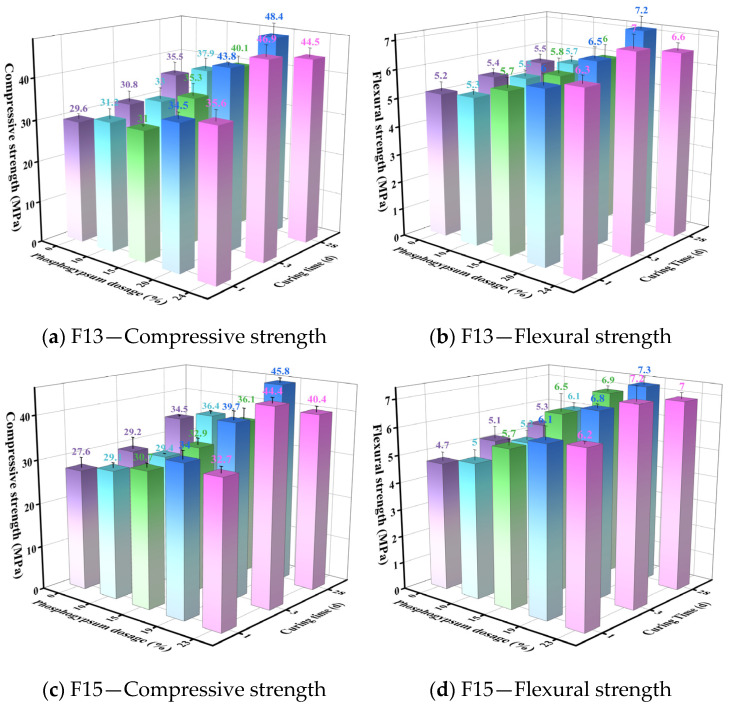
Effect of phosphogypsum dosage on the strength of SW-SAC.

Clinkers produced from F13 (1350 °C, 30 min) and F15 (1350 °C, 20 min) were selected to prepare SW-SAC pastes with varying phosphogypsum dosages according to the mix proportions in Table 3. The measured compressive and flexural strengths are presented in Figure 12.

As shown in Figure 12, increasing the phosphogypsum (PG) dosage promotes early-age strength (1 and 3 d), whereas the 28 d strength increases initially and then declines at higher PG levels. For the F13 series, the 28 d strength reached its maximum at 20 wt% PG, with compressive and flexural strengths of 48.4 MPa and 7.2 MPa, respectively. When the PG dosage was further increased to 24 wt%, early-age strength improved slightly, yet the 28 d strength dropped markedly to 44.5 MPa (compressive) and 6.6 MPa (flexural). A similar trend was observed for the F15 series, where the optimal PG dosage was 19 wt% and the corresponding 28 d compressive and flexural strengths were 45.8 MPa and 7.3 MPa. These results indicate that an appropriate PG addition can favorably regulate hydration and strength development of sulfoaluminate cement, while excessive PG becomes detrimental to later-age performance. Under the present experimental conditions, the optimal PG dosages were 20 wt% for F13 and 19 wt% for F15.

This dosage-dependent behavior is closely linked to changes in the hydration products of ye’elimite ($C_{4}A_{3}\bar{S}$) as the PG level varies. At 10 wt% PG, the gypsum coefficient *M* is 0.7 and AFm is the predominant hydration product of $C_{4}A_{3}\bar{S}$. Raising the PG dosage to 15 and 20 wt% increases *M* to 1.1 and 1.5, respectively; under these conditions, AFm and AFt coexist, with AFt progressively increasing and AFm decreasing as PG rises. Because AFt formation involves volumetric expansion, it can enhance matrix densification during early hardening, which is generally beneficial for strength development. At 24 wt% PG (*M* = 1.9), hydration of $C_{4}A_{3}\bar{S}$ yields predominantly AFt, with only a small amount of AFm remaining. At early ages (1 and 3 d), the expansion-driven pore filling and improved interparticle bonding associated with AFt formation may still contribute to continued strength gain. By 28 d, however, the abundance of AFt could potentially give rise to excessive expansion-induced internal stresses and microcracking within the hardened matrix, thereby compromising the later-age strength. Bizzozero et al. [52] demonstrated that CSA systems exhibit a critical calcium sulfate content, above which unstable expansion and failure may occur, and attributed this behavior to increased supersaturation with respect to ettringite and the resulting crystallization pressure in the pore network. Chen et al. [57] further showed that the expansion of calcium sulfoaluminate–belite cements is strongly affected by calcium sulfate dosage, together with other factors such as ye’elimite content, water-to-cement ratio, and particle fineness, and that ettringite formation may lead to expansive cracking under certain conditions. In addition, Beltagui et al. [58] reported that gypsum and water contents significantly influence the hydration, pore structure, and compressive strength of belite-calcium sulphoaluminate cement, with a non-linear dependence of strength on gypsum content. These studies support the interpretation that the strength reduction observed at 24 wt% PG (M = 1.9) may be associated with excessive sulfate availability, enhanced AFt formation, and the resulting risk of expansion-induced internal damage. However, since direct microstructural evidence of microcracking was not obtained in the present study, this explanation should be regarded as a literature-supported interpretation rather than direct proof.

#### 3.2.2. Effects of Phosphorus Slag and Limestone Powder (Supplementary Materials) on SW-SAC Performance

**Figure 13:**
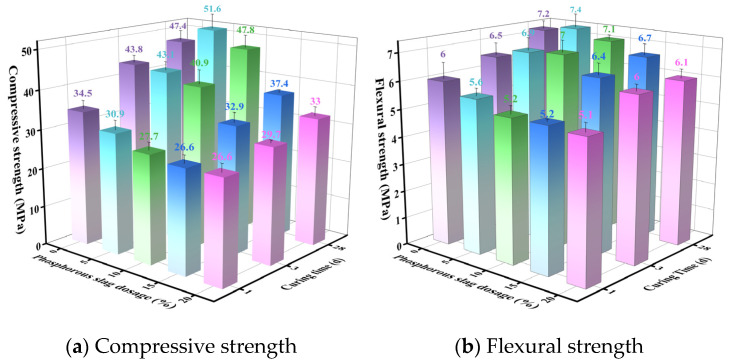
Effect of phosphorus slag dosage on the strength of SW-SAC.

**Figure 14:**
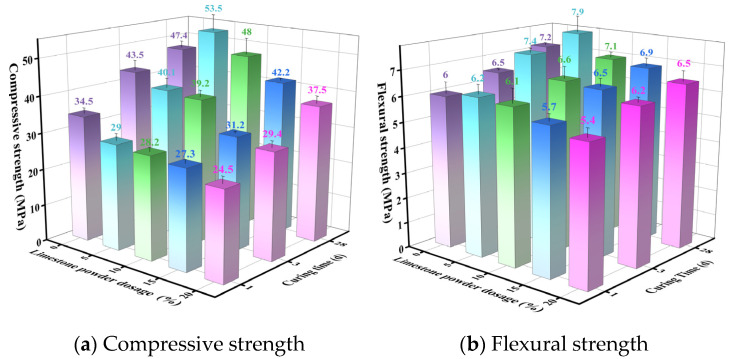
Effect of limestone powder dosage on the strength of SW-SAC.

Using the F13-PG20 mixture as the reference, phosphorus slag powder and limestone powder were introduced as Supplementary materials to evaluate how their dosages affect the strength of SW-SAC. The mix proportions are given in Table 4, and the corresponding test results are presented in Figure 13 and Figure 14.

Figure 13 shows that incorporating phosphorus slag led to a progressive reduction in 1 and 3 d compressive strength as well as 1 d flexural strength. This response is mainly associated with the low early reactivity of phosphorus slag and its tendency to retard hydration at early ages [59]. In contrast, the 3 d flexural strength and the 28 d compressive and flexural strengths increased initially with slag addition and then declined, reaching their maxima at 5 wt% slag; at this dosage, the 28 d compressive and flexural strengths were 51.6 MPa and 7.4 MPa, respectively. This improvement at later ages can be attributed to the progressive hydration of belite (C_2_S), which increases the availability of Ca(OH)_2_ and thereby activates secondary reactions of phosphorus slag, producing additional hydrates—most notably C–S–H gel—that contribute to the 28 d strength [60]. A similar dilution-then-compensation pattern has been reported by García-Maté et al. [61] for CSA cements blended with fly ash, where moderate SCM incorporation reduced early-age strength but enhanced later-age strength through secondary hydrate formation, in good agreement with the trend observed in this study. Once the slag dosage became relatively high (≥10 wt%), the clinker fraction decreased substantially, limiting the overall amount of hydrates formed; consequently, the 28 d strength showed an overall downward trend.

As indicated by Figure 14, limestone powder exhibits a similar influence on compressive strength: the 1 and 3 d values decreased gradually as the limestone dosage increased, whereas the 28 d compressive strength rose initially and then fell. The maximum 28 d compressive strength (53.5 MPa) occurred at 5 wt% limestone, slightly exceeding the value obtained at the same phosphorus slag dosage (51.6 MPa). Flexural strength at 1, 3, and 28 d followed a rise–fall pattern with increasing limestone content, peaking at 5 wt% with values of 6.2, 7.4, and 7.9 MPa, respectively. On the one hand, limestone acts as an inert filler, and increasing its dosage inevitably dilutes the clinker phases, which tends to impair early compressive strength. On the other hand, limestone can exhibit a moderate reactive effect by participating in hydration and forming monocarboaluminate (C_3_A·CaCO_3_·11H_2_O), which may support later-age strength development [62]. The 10.5% gain in 28 d compressive strength obtained at only 5 wt% limestone substitution is consistent with the ettringite-stabilization and carboaluminate-formation mechanisms documented for CSA–limestone systems by Pelletier–Chaignat [63]. When the limestone dosage becomes excessive, dilution of the clinker phases limits hydrate formation, and the strength correspondingly decreases. This interpretation is further supported by the XRD and TG–DTG results discussed later.

### 3.3. Hydration of SW-SAC

To investigate the hydration behavior of SW-SAC, cement pastes were prepared according to the mix proportions listed in Table 10.

#### 3.3.1. XRD Analysis of Hydration Products

**Figure 15:**
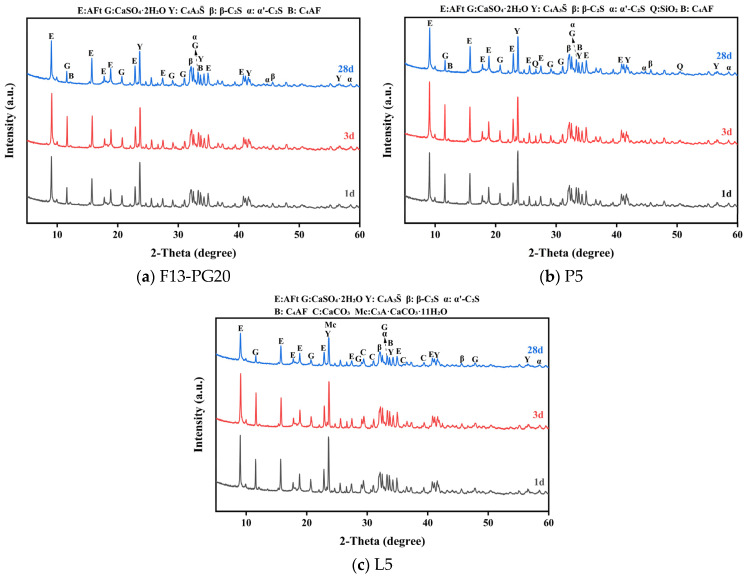
XRD patterns of hydrated SW-SAC pastes with different mix designs at various curing ages.

Figure 15 shows the XRD patterns of hydrated paste samples (1, 3, and 28 d) for the three cements listed in Table 10. From Figure 15a, the hydrated F13-PG20 paste (20 wt% phosphogypsum) is characterized by ettringite (AFt) as the main hydrate, together with residual unreacted phases including ye’elimite ($C_{4}A_{3}\bar{S}$), β-C_2_S, α′-C_2_S, C_4_AF, and gypsum. With increasing curing age from 1 to 3 and 28 d, the characteristic reflections of $C_{4}A_{3}\bar{S}$ progressively weaken. Distinct AFt peaks are already evident at 1 d and intensify markedly by 3 d, whereas the AFt peak intensity at 28 d is slightly lower than that at 3 d. These observations indicate that, once mixed with water, SW-SAC undergoes rapid and sustained hydration of $C_{4}A_{3}\bar{S}$, leading to a gradual decrease in its residual content with time. During the early stage (1–3 d), rapid AFt formation from $C_{4}A_{3}\bar{S}$ is consistent with the pronounced strength gain observed over the same period (Figure 12), underscoring the critical contribution of ettringite to early-age strength development in sulfoaluminate cement. At 28 d, the hydrate assemblage evolves further, accompanied by an increased presence of gel-type hydrates, which supports continued strength development.

Figure 15b,c present the XRD patterns of hydrated pastes for mix P5 (5 wt% phosphorus slag) and mix L5 (5 wt% limestone powder) at different curing ages, respectively. The incorporation of phosphorus slag or limestone powder does not markedly alter the types of the main hydration products, nor the overall evolution trend of their intensities. A notable difference is observed for P5, where the AFt reflections at 1 and 3 d are weaker than those of the slag-free reference (P0). This can be attributed to soluble phosphorus species in the slag (e.g., H_3_PO_4_, H_2_PO_4_^−^, and HPO_4_^2−^), which react with Ca^2+^ to form insoluble calcium phosphates such as Ca_3_(PO_4_)_2_. These precipitates can deposit on clinker particle surfaces and form a passivating layer, limiting further hydration of ye’elimite ($C_{4}A_{3}\bar{S}$) and consequently delaying AFt formation [55,58]. In contrast, the limestone-containing paste (L5) exhibits monocarboaluminate (Mc) as an identifiable hydration product. This behavior is consistent with sulfoaluminate hydration under a gypsum coefficient M < 2, where the available CaSO_4_ is insufficient to drive complete conversion of $C_{4}A_{3}\bar{S}$ into AFt. Under such conditions, CaCO_3_ from limestone can partially substitute for CaSO_4_ and react with Al-bearing phases—primarily C_3_A and $C_{4}A_{3}\bar{S}$—to form monocarboaluminate (Mc) [60]. Moreover, by consuming part of the available aluminate, CaCO_3_ can suppress the conversion of AFt to AFm, thereby stabilizing AFt. The associated increase in total hydrate volume can improve matrix compactness and, consequently, mechanical performance [60,63,64].

#### 3.3.2. TG–DTG Analysis

**Figure 16:**
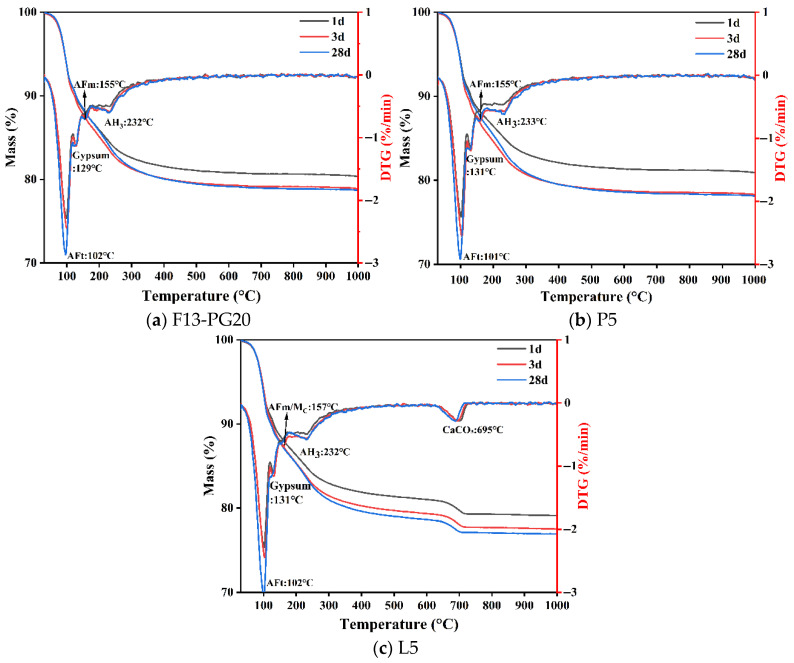
TG/DTG curves of hydrated SW-SAC pastes with different mix designs at various curing ages.

Figure 16 shows the TG–DTG curves of hydrated samples for the three SW-SAC pastes listed in Table 10. In thermogravimetric analyses of cementitious materials, the mass-loss peak around 80–120 °C is commonly assigned to dehydration of AFt [65], while the 120–145 °C interval is closely associated with gypsum dehydration [11]. The broader mass loss from 120 to 260 °C is mainly attributed to dehydration of monocarboaluminate (Mc), within which the 145–180 °C range can be related to AFm dehydration/decomposition [66]. In addition, the continuous mass loss over 50–1000 °C largely reflects the gradual release of bound water from C–S–H gel [67], and the 200–300 °C region mainly corresponds to dehydroxylation of AH_3_ [68]. The mass-loss event near 700 °C can be attributed to thermal decomposition of CaCO_3_ in the hydrated samples [69]. Because individual hydrates exhibit characteristic decomposition ranges, the magnitude of mass loss within the same temperature intervals can be used to qualitatively compare the relative amounts of the corresponding hydration products among different samples. Table 11 summarizes the mass-loss values of the three hydrated samples within these characteristic temperature intervals.

Figure 16 and Table 11 show that, as hydration proceeds from 1 to 28 d, the mass loss of the F13-PG20 sample in the 80–120 °C range first increases and then decreases slightly, in agreement with the XRD observations (Figure 15). This behavior suggests that AFt forms rapidly at early ages and then stabilizes, with only limited transformation at later stages. The progressive reduction in mass loss within 120–145 °C confirms continuous gypsum consumption during hydration. A modest increase in the mass loss assigned to AFm dehydration (145–180 °C) indicates a slight rise in AFm content as hydration advances. Meanwhile, the mass loss associated with dehydration of C–S–H gel and dehydroxylation of AH_3_ becomes more pronounced with curing time, implying continuous formation of these gel-type products, which contribute to microstructural densification and strength development. It should be noted that characteristic reflections of AFm and AH_3_ are not clearly identifiable in the XRD patterns (Figure 15), which is likely due to the low content and poor crystallinity of AFm as well as the predominantly amorphous nature of AH_3_.

For P5, the mass loss in the 80–120 °C interval shows an increase followed by a slight decrease with curing time, yet it remains lower than that of F13-PG20 at the same ages. Meanwhile, the mass loss associated with gypsum dehydration (120–145 °C) is relatively higher, collectively indicating that phosphorus slag addition reduces the amount of AFt formed. In L5, the AFt-related mass loss increases steadily with age, suggesting that limestone powder can inhibit the conversion of AFt to AFm and thereby enhance AFt stability [63,70,71]. Notably, the 28 d AFt-related mass loss of L5 (7.68 wt%) exceeds that of P5 (7.23 wt%), further confirming that limestone powder is more effective in preserving ettringite. In parallel, the contents of gypsum and CaCO_3_ decrease with time, consistent with their consumption during the formation of AFt and monocarboaluminate (Mc), respectively [60]. To further quantify the effects of the two mineral additions, the total gel-phase bound water—defined as the combined mass losses from C–S–H dehydration and AH_3_ dehydroxylation—was compared at 28 d. The values are 10.20 wt% for P5, 9.79 wt% for L5, and 9.10 wt% for F13-PG20, indicating that both mineral additions enhance the overall formation of gel-type hydrates relative to the unadmixed baseline. Between the two, phosphorus slag yields a greater quantity of gel-phase products, whereas limestone powder preferentially promotes ettringite formation and stabilization. Such enhancement can be attributed to the secondary hydration of phosphorus slag in P5 and, for L5, to the accelerating and reactive effects of limestone powder on sulfoaluminate hydration [68,69].

#### 3.3.3. SEM Analysis

**Figure 17:**
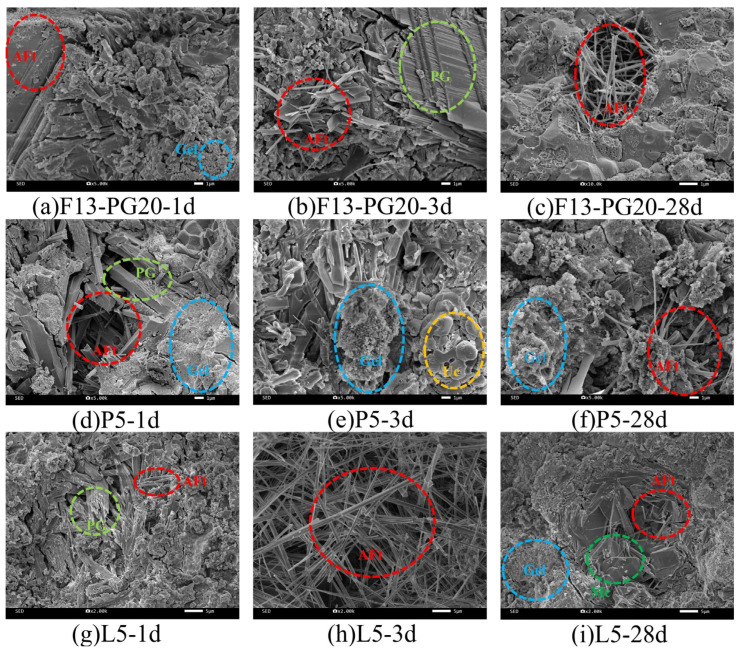
SEM images of hydrated SW-SAC pastes with different mix designs at various curing ages.

The SEM images of the hydrated SW-SAC pastes are presented in Figure 17 to corroborate the hydrate evolution inferred from XRD and TG–DTG analyses.

As shown in Figure 17a–c, the F13-PG20 group at 1 d is dominated by fine needle-like AFt crystals with a loose and porous structure. With increasing curing age, the AFt crystals become progressively coarser and more abundant, interweaving with each other, while gel products fill the interstices between crystals, resulting in a gradually denser overall structure. As shown in Figure 17d–f, the P5 group at 1 d exhibits short and sparsely distributed AFt crystals, with noticeably less AFt formation compared to the F13-PG20 group. This is attributed to the complexation of P_2_O_5_ from phosphorus slag with Ca^2+^/Al^3+^, which inhibits early AFt growth. With prolonged curing, the AFt crystals gradually develop into coarser forms and interconnect into a network. Meanwhile, the reactive SiO_2_ in phosphorus slag participates in hydration reactions, generating a considerable amount of gel products that effectively fill the pores, leading to a significant improvement in densification. As shown in Figure 17g–i, the L5 group at 1 d is mainly composed of fine needle-like AFt crystals with a loose structure. As curing age increases, AFt crystals grow extensively and interweave, while plate-like Mc crystals are interspersed among them and gel products fill the interstices, resulting in a progressively denser structure. The presence of carbonate ions in the system promotes the preferential formation of Mc from part of the aluminate phases, thereby indirectly stabilizing the AFt phase.

### 3.4. Carbon Footprint and Economic Viability Analysis of SW-SAC

Taking SW-SAC produced from the F13 clinker as a representative case, a life cycle assessment (LCA) approach was applied to quantify CO_2_ emissions across the full production chain [72]. The system boundary covered raw material extraction, transportation, and the “two grinding and one calcination” process, including direct emissions (notably those associated with fuel consumption) as well as indirect emissions arising from electricity use. Using China’s energy mix data and values reported in the literature, emission factors were employed to estimate the carbon footprint of each production stage. The results were then benchmarked against ordinary Portland cement (OPC; P·I) and conventional sulfoaluminate cement (SAC) to evaluate the environmental benefits and mitigation potential of producing SW-SAC from industrial residues [73]. The calculation framework is given in Equations (14)–(19), and the raw material consumption values used for the LCA are summarized in Table 12.

(14) $$M_{1}=M_{crm}\times \left(1-W_{sw}\right)\times E_{0}\times E_{grid,OM\;Simple,y}$$

(15) $$M_{2}=M_{crm}\times E_{do}\times E_{co_{2},do,heat}$$

(16) $$M_{3}=\frac{44}{56}\times \gamma_{CaO}+\frac{44}{40}\times \gamma_{MgO}$$

(17) $$M_{4}=M_{c}\times E_{co_{2},e,heat}$$

(18) $$M_{5}=M_{e}\times E_{grid,OM\;Simple,y}$$

(19) $$M=M_{1}+M_{2}+M_{3}+M_{4}+M_{5}$$

where *M*_1_ is the carbon footprint associated with raw material extraction (kg); *M_crm_* is the amount of raw meal required per unit mass of cement clinker (kg); *W_sw_* is the solid-waste utilization ratio (%); and E_0_ is the electricity demand for raw material extraction (kWh/kg), taken as E0 = 0.014 kWh/kg [61]. *E_grid,OMSimple,y_* denotes the marginal emission factor of the regional power grid (kg/kWh), set to 0.5271 kg/kWh [74]. *M*_2_ represents the carbon footprint from raw material transportation (kg); *E_do_* is the diesel consumption rate of a fully loaded truck (L/km); and $E_{co_{2},e,heat}$ is the diesel emission factor based on lower heating value, taken as 3.67 kg/L [75]. *M*_3_ is the carbon footprint arising from CO_2_ released by raw meal decomposition during calcination (kg); $\gamma_{\mathrm{CaO}}$ and $\gamma_{\mathrm{MgO}}$ are the mass fractions of CaO and MgO in the clinker (%), and 44/56 and 44/40 are the molecular-weight ratios of CO2 to CaO and MgO, respectively. *M*_4_ is the carbon footprint associated with coal consumption during cement production (kg); *M_c_* is the coal consumption (kg); and $E_{co_{2},e,heat}$ is the coal emission factor (kg/kg), taken as 2.83 kg/kg [76]. Finally, *M*_5_ denotes the carbon footprint attributable to electricity consumption during cement production (kg), where *M_e_* is the electricity use (kWh).

Given that phosphogypsum, calcium carbide residue, and red mud are industrial by-products, their extraction-stage burdens were not accounted for; consequently, *M*_1_ includes only the emissions associated with bauxite mining. For the transportation stage, a 5 t diesel truck was assumed with a hauling distance of 100 km and a fuel consumption rate of 0.2 L/km under full load [73], from which *M*_2_ was calculated. Because calcium carbide residue, phosphogypsum, red mud, and bauxite were assumed not to undergo carbonate decomposition under the idealized conditions considered here, *M*_3_ was set to zero for the F13-based system. Coal consumption and baseline electricity use were taken from Ref. [77]: the coal demand for P·I, SAC, and F13 was 125, 118, and 115 kg per ton of cement, respectively, and the corresponding electricity consumption was 107, 104, and 95 kWh, which were used to obtain *M*_4_ and *M*_5._ The resulting carbon footprints calculated using Equations (14)–(19) are summarized in Table 13. Relative to P·I and conventional SAC, the life-cycle carbon footprint decreases by 59.6% and 41.9%, respectively, highlighting the substantial mitigation potential of synergistic solid-waste utilization combined with process optimization.

To evaluate the economic viability, the production costs of F13-based SW-SAC, P·I cement, and conventional SAC were compared based on raw material cost, transport cost, coal consumption, and electricity cost. Phosphogypsum, carbide slag, and red mud are classified as industrial solid wastes and therefore incur zero material cost. The unit prices of all raw materials and energy inputs are summarized in Table 14, and the comparative results are presented in Table 15.

As shown in Table 15, the production cost of F13 is approximately 520 CNY/t, 39.8% lower than conventional SAC (≈862 CNY/t), owing to the replacement of bauxite and natural gypsum with low-cost industrial wastes. Although higher than P·I cement (≈271 CNY/t), the 59.6% CO_2_ reduction offers potential revenue under China’s Emissions Trading Scheme, and government incentives for waste utilization further narrow the cost gap.

## 4. Conclusions

This study produced sulfoaluminate cement (SW-SAC) from a raw meal in which industrial solid wastes—phosphogypsum, calcium carbide residue, and red mud—account for approximately 75% of the total mass, with calcium carbide residue replacing limestone as the primary CaO source and phosphogypsum supplying SO_3_ via single-step direct calcination. The effects of calcination temperature and holding time on clinker phase assemblage and performance were systematically evaluated. Phosphogypsum was then used as a setting regulator, and phosphorus slag or limestone powder was incorporated as Supplementary materials to prepare SW-SAC meeting the 42.5 strength class. The hydration process and microstructural evolution were characterized by XRD, TG–DTG, and SEM. A carbon footprint assessment and production cost analysis were also conducted. The main conclusions are summarized as follows.

(1) Raw meals comprising phosphogypsum, calcium carbide residue, red mud, and bauxite were proportioned according to stoichiometrically balanced formulations and subjected to calcination at 1350 °C with a controlled isothermal hold of 20–30 min. This yielded SW-SAC clinker in which $C_{4}A_{3}\bar{S}$ and C_2_S constitute the dominant crystalline phases. SEM–EDS observations further confirmed that the characteristic $C_{4}A_{3}\bar{S}$ crystals were well developed, exhibiting regular plate-like morphologies. Quantitative XRD analysis confirmed that, under the established process conditions, the clinker contains 58–64 wt% $C_{4}A_{3}\bar{S}$ and 25–29 wt% C_2_S. $C_{4}A_{3}\bar{S}$ is predominantly present in the orthorhombic polymorph, while a minor fraction—approximately 14–16 wt%—exists as the metastable cubic polymorph ($C_{4}A_{3}\bar{S}-c$).

(2) Blending 20 wt% phosphogypsum into SW-SAC clinker yielded sulfoaluminate cement conforming to the GB/T 20472-2006 [85] strength class 42.5, with compressive strengths of 34.5 MPa (1 d), 43.8 MPa (3 d), and 48.4 MPa (28 d). Subsequent incorporation of 5 wt% phosphorus slag or limestone powder—as supplementary cementitious materials—led to enhanced late-age strength. Specifically, the phosphorus slag-blended SW-SAC exhibited compressive strengths of 30.9 MPa (1 d), 43.1 MPa (3 d), and 51.6 MPa (28 d), representing a 6.7% increase at 28 d relative to the phosphogypsum-only reference. The limestone-blended variant achieved 29.0 MPa (1 d), 40.1 MPa (3 d), and 53.5 MPa (28 d), corresponding to a 10.5% gain at 28 d.

(3) Microstructural analysis confirms that the three SW-SAC formulations produced in this study consistently yield ettringite (AFt), aluminum hydroxide gel (AH_3_), and calcium silicate hydrate (C–S–H) gel as the predominant hydration products under standard curing conditions (20 °C, ≥95% RH). Upon incorporation of 5 wt% phosphorus slag or limestone powder, distinct mechanisms drive enhanced hydration: phosphorus slag undergoes pozzolanic reaction with portlandite (Ca(OH)_2_), generating supplementary C–S–H; limestone facilitates carbonate ion exchange with sulfate-bearing AFt, promoting the formation of monocarboaluminate (AFm). Both mechanisms accelerate SW-SAC hydration, leading to a measurable increase in C–S–H gel content at 28 days—directly contributing to the observed enhancement in compressive strength.

(4) The LCA demonstrates that the carbon footprint of SW-SAC production is 426.2 kg CO_2_-eq/t, representing a 41.9% reduction compared with conventional SAC (733.8 kg CO_2_-eq/t) and a 59.6% reduction relative to P·I cement (1054.1 kg CO_2_-eq/t). Moreover, the production cost of F13 is approximately 520 CNY/t, 39.8% lower than conventional SAC (≈862 CNY/t), owing to the substitution of bauxite and natural gypsum with low-cost industrial wastes. Although higher than P·I cement (≈271 CNY/t), the substantial CO_2_ reduction offers potential revenue under China’s Emissions Trading Scheme, and government incentives for waste utilization further narrow the cost gap. These results suggest that SW-SAC may serve as a viable low-carbon alternative, although pilot-scale validation and long-term durability testing are still required.

## Figures and Tables

**Table 1:** Major oxide compositions of the raw materials (wt%).

Raw Material	CaO	SiO_2_	Al_2_O_3_	Fe_2_O_3_	SO_3_	TiO_2_	P_2_O_5_	F	MgO	LOI
Phosphogypsum	27.72	6.77	0.59	0.35	40.84	0.13	0.97	1.49	n.d.	20.23
Calcium carbide residue	67.97	3.59	1.54	0.12	0.46	-	-	-	-	25.27
Red mud	1.82	14.79	21.08	52.99	1.02	6.39	-	-	-	-
Bauxite	0.77	17.58	73.09	3.82	0.06	3.43	-	-	-	0.28
Phosphorus slag	50.30	37.80	3.30	-	1.10	-	2.40	-	0.5	−0.27
Limestone powder	53.75	1.33	0.10	-	-	-	-	-	1.20	41.66

Note: “-” indicates that the component was not detected by XRF; the negative LOI of phosphorus slag (−0.27%) is attributed to the oxidation of low-valence oxides (e.g., Fe_3_O_4_ → Fe_2_O_3_) within its glassy phase during ignition, resulting in a net mass gain.

**Table 2:** Designed clinker mineral composition, characteristic ratios, and raw meal proportions of SW-SAC.

Mix ID	Designed Clinker Mineral Composition (wt%)			Clinker Characteristic Ratios			Raw Meal Proportions (wt%)			
	$C_{4}A_{3}\bar{S}$	C_2_S	C_4_AF	C_m_	P	N	PG	CCR	RM	BX
F13	52.00	30.00	13.00	0.99	3.42	2.47	14.95	50.02	4.37	30.66
F15	49.00	30.00	15.00	0.99	3.47	2.39	14.14	50.61	5.52	29.73

**Table 3:** Mix proportions of SW-SAC cement with different phosphogypsum contents (wt%).

Mix ID	Gypsum Coefficient, *M*	Clinker (wt%)	Phosphogypsum (wt%)
F13-PG0	0	100	0
F13-PG10	0.7	90	10
F13-PG15	1.1	85	15
F13-PG20	1.5	80	20
F13-PG24	1.9	76	24
F15-PG0	0	100	0
F15-PG10	0.7	90	10
F15-PG15	1.1	85	15
F15-PG19	1.5	81	19
F15-PG23	1.9	77	23

**Table 4:** Mix compositions of SW-SAC with different dosages of phosphorus slag or limestone powder (wt%).

Mix ID	SW-SAC Clinker (wt%)	Phosphogypsum (wt%)	Phosphorus Slag/Limestone Powder
P0/L0	80	20	0/0
P5/L5	76	19	5/5
P10/L10	72	18	10/10
P15/L15	68	17	15/15
P20/L20	64	16	20/20

Note: The value before “/” denotes the dosage of phosphorus slag in the single-addition series (P series), whereas the value after “/” denotes the dosage of limestone powder in the single-addition series (L series).

**Table 5:** Crystal structures of the phases involved in RQPA.

Phase	Space Group	ICSD Code	Phase	Space Group	ICSD Code
C_4_A_3_S-o	Pcc2	80361	C_4_A_3_S-c	I4-3m	28480
β-C_2_S	P121/n1	81096	α′-C_2_S	Pnma	81097
C_4_AF	Pnma	45646	C_3_A	Pa3-	51369
C_12_A_7_	I4-3d	41241	CT	Pnma	77060
CaO	Fm3-m	28905			

**Table 6:** XRD–Rietveld quantitative phase analysis of F13 clinkers calcined at different temperatures (wt%).

	$C_{4}A_{3}\bar{S}\text{-}o$	$C_{4}A_{3}\bar{S}\text{-}c$	β-C_2_S	α′-C_2_S	C_4_AF	C_3_A	C_12_A_7_	CT	f-CaO	Rwp
1300 °C	48.44	13.49	29.60	1.09	1.92	2.09	0.52	2.86	-	8.44
1325 °C	47.30	13.03	29.36	1.21	1.86	2.50	1.85	2.91	-	8.42
1350 °C	49.16	14.90	24.23	0.82	2.31	2.29	3.35	2.91	0.03	9.09
1375 °C	45.35	11.75	28.50	-	1.69	2.14	5.85	4.74	-	7.59
1400 °C	42.25	9.76	29.35	-	1.94	2.38	8.64	5.68	-	7.34

**Table 7:** XRD–Rietveld quantitative phase analysis of F15 clinkers calcined at different temperatures (wt%).

	$C_{4}A_{3}\bar{S}\text{-}o$	$C_{4}A_{3}\bar{S}\text{-}c$	β-C_2_S	α′-C_2_S	C_4_AF	C_3_A	C_12_A_7_	CT	f-CaO	Rwp
1300 °C	48.77	10.42	10.55	19.58	6.91	1.38	1.35	0.59	0.46	8.32
1325 °C	47.55	11.24	18.89	11.49	7.44	1.84	1.12	0.34	0.09	8.13
1350 °C	45.69	14.27	24.39	1.01	3.05	1.72	5.47	4.37	0.04	7.92
1375 °C	43.07	8.98	30.06	1.80	5.87	2.17	7.86	0.19	-	7.36
1400 °C	39.76	8.66	25.03	9.24	4.25	3.91	7.03	2.14	-	7.02

**Table 8:** XRD–Rietveld quantitative phase analysis of F13 clinkers held at 1350 °C for different durations (wt%).

	$C_{4}A_{3}\bar{S}\text{-}o$	$C_{4}A_{3}\bar{S}\text{-}c$	β-C_2_S	α′-C_2_S	C_4_AF	C_3_A	C_12_A_7_	CT	f-CaO	Rwp
10 min	46.47	14.38	17.81	14.41	4.07	1.39	0.92	0.12	0.01	9.15
20 min	42.55	16.43	17.73	15.80	2.81	2.50	0.76	0.36	-	8.95
30 min	49.16	14.90	24.23	0.82	2.31	2.29	3.35	2.91	0.03	9.09
40 min	41.78	17.35	17.11	12.66	2.14	2.84	4.71	0.58	-	9.74
50 min	42.08	16.07	13.36	15.18	2.54	2.54	6.86	0.46	-	9.24

**Table 9:** XRD–Rietveld quantitative phase analysis of F15 clinkers held at 1350 °C for different durations (wt%).

	$C_{4}A_{3}\bar{S}\text{-}o$	$C_{4}A_{3}\bar{S}\text{-}c$	β-C_2_S	α′-C_2_S	C_4_AF	C_3_A	C_12_A_7_	CT	f-CaO	Rwp
10 min	41.71	14.57	19.38	12.55	8.33	1.83	0.90	0.29	0.12	9.69
20 min	42.15	15.97	16.53	12.96	6.14	3.41	1.11	0.82	-	9.28
30 min	45.69	14.27	24.39	1.01	3.05	1.72	5.47	4.37	0.04	7.92
40 min	36.24	16.82	14.78	10.26	6.48	3.76	5.85	5.26	-	8.99
50 min	35.04	17.33	15.86	14.86	6.93	3.23	5.08	0.89	-	8.88

**Table 10:** Optimized mix proportions of SW-SAC used for hydration analysis (wt%).

Mix ID	F13 Clinker	Phosphogypsum	Phosphorus Slag	Limestone Powder
F13-PG20	80%	20%	-	-
P5	76%	19%	5%	-
L5	76%	19%	-	5%

**Table 11:** TG–DTG mass loss of hydrated sulfoaluminate cement pastes in selected temperature ranges (wt%).

Temperature Range (Assignment)	F13-PG20			P5			L5		
	1 d	3 d	28 d	1 d	3 d	28 d	1 d	3 d	28 d
80–120 °C (AFt dehydration)	7.73	8.28	7.87	6.70	7.70	7.23	6.75	7.36	7.68
120–145 °C (gypsum dehydration)	2.20	2.16	2.05	2.19	2.31	2.17	2.60	2.29	2.04
145–180 °C (AFm dehydration)	1.79	1.98	1.99	1.66	2.07	2.06	1.71	1.96	1.87
200–300 °C (AH_3_ dehydroxylation)	3.33	3.82	4.34	3.28	3.89	4.47	3.33	3.87	4.28
640–730 °C (CaCO_3_ decomposition)	-	-	-	-	-	-	1.58	1.48	1.39
50–1000 °C (C–S–H dehydration)	4.19	4.44	4.76	4.90	5.35	5.73	4.54	5.17	5.51

**Table 12:** Raw material consumption for producing 1 t of cement: F13-based SW-SAC vs. P·I and SAC.

Cement Type	Limestone	BX	Clay	Natural Gypsum	CCR	PG	RM	Total Raw Meal
F13	-	291	-	-	475	356	42	1164
P·I	1300	-	300	50	-	-	-	1650
SAC	635	494	-	282		-	-	1411

**Table 13:** Unit carbon footprint by production stage for F13, P·I, and SAC (kg CO_2_/t cement).

Cement Type	Raw Material Extraction, M_1_	Raw Material Transport, M_2_	Carbonate Decomposition, M_3_	Fuel Combustion, M_4_	Electricity Use, M_5_	Total, M
F13	2.1	17	0	325.5	81.6	426.2
P·I	12.2	24.2	572.0	353.8	91.9	1054.1
SAC	10.4	20.8	279.4	333.9	89.3	733.8

**Table 14:** Unit prices of raw materials and energy.

Raw Material	Type	Price	Source
PG	Solid waste	0 CNY/t	MEE Catalog (2024) [78]
CCR	Solid waste	0 CNY/t	MEE Catalog (2024)
RM	Solid waste	0 CNY/t	MEE Catalog (2024)
BX	Mineral	1250 CNY/t	Mysteel (2025) [79]
Limestone	Mineral	55 CNY/t	HERI (2025) [80]
Clay	Mineral	40 CNY/t	Industry average
Natural gypsum	Mineral	150 CNY/t	Ccement.com [81]
Coal (5000 kcal)	Energy	750 CNY/t	BJX Energy (2025) [82]
Electricity (industrial)	Energy	0.45 CNY/kWh	Hubei DRC (2025) [83]
Road transport	Logistics	0.23 CNY/t·km	CFLP (2024) [84]

MEE = Ministry of Ecology and Environment; HERI = Huajing Industry Research Institute; CFLP = China Federation of Logistics and Purchasing; BJX = BJX Energy News; Hubei DRC = Hubei Development and Reform Commission.

**Table 15:** Comparative production cost per ton of clinker meal (CNY).

Cement Type	Material Cost (CNY/t)	Transport Cost (CNY/t)	Energy Cost (CNY/t)	Total Cost (CNY/t)
F13	363.75	26.78	129.00	519.53
P·I	91.00	37.95	141.90	270.85
SAC	694.73	32.46	135.30	862.49

## Data Availability

The original contributions presented in this study are included in the article. Further inquiries can be directed to the corresponding authors.
